# Neuroprotective Effects of Exercise Postconditioning After Stroke *via* SIRT1-Mediated Suppression of Endoplasmic Reticulum (ER) Stress

**DOI:** 10.3389/fncel.2021.598230

**Published:** 2021-02-16

**Authors:** Fengwu Li, Xiaokun Geng, Hangil Lee, Melissa Wills, Yuchuan Ding

**Affiliations:** ^1^China-America Institute of Neuroscience, Luhe Hospital, Capital Medical University, Beijing, China; ^2^Department of Neurology, Beijing Luhe Hospital, Capital Medical University, Beijing, China; ^3^Department of Neurosurgery, Wayne State University School of Medicine, Detroit, MI, United States; ^4^Department of Research and Development Center, John D. Dingell VA Medical Center, Detroit, MI, United States

**Keywords:** ischemia/reperfusion, exercise intensity, ER stress, apoptosis, caspase-12, CHOP

## Abstract

While it is well-known that pre-stroke exercise conditioning reduces the incidence of stroke and the development of comorbidities, it is unclear whether post-stroke exercise conditioning is also neuroprotective. The present study investigated whether exercise postconditioning (PostE) induced neuroprotection and elucidated the involvement of SIRT1 regulation on the ROS/ER stress pathway. Adult rats were subjected to middle cerebral artery occlusion (MCAO) followed by either: (1) resting; (2) mild exercise postconditioning (MPostE); or (3) intense exercise postconditioning (IPostE). PostE was initiated 24 h after reperfusion and performed on a treadmill. At 1 and 3 days thereafter, we determined infarct volumes, neurological defects, brain edema, apoptotic cell death through measuring pro- (BAX and Caspase-3) and anti-apoptotic (Bcl-2) proteins, and ER stress through the measurement of glucose-regulated protein 78 (GRP78), inositol-requiring 1α (IRE1α), protein kinase RNA-like endoplasmic reticulum kinase (PERK), activating transcription factor 6 (ATF6), C/EBP homologous protein (CHOP), Caspase-12, and SIRT1. Proteins were measured by Western blot. ROS production was detected by flow cytometry.Compared to resting rats, both MPostE and IPostE significantly decreased brain infarct volumes and edema, neurological deficits, ROS production, and apoptotic cell death. MPostE further increased Bcl-2 expression and Bcl-2/BAX ratio as well as BAX and Caspase-3 expressions and ROS production (*p < 0.05). Both PostE groups saw decreases in ER stress proteins, while MPostE demonstrated a further reduction in GRP78 (***p < 0.001) and Caspase-12 (*p < 0.05) expressions at 1 day and IRE1α (**p < 0.01) and CHOP (*p < 0.05) expressions at 3 days. Additionally, both PostE groups saw significant increases in SIRT1 expression.In this study, both mild and intense PostE levels induced neuroprotection after stroke through SIRT1 and ROS/ER stress pathway. Additionally, the results may provide a base for our future study regarding the regulation of SIRT1 on the ROS/ER stress pathway in the biochemical processes underlying post-stroke neuroprotection. The results suggest that mild exercise postconditioning might play a similar neuroprotective role as intensive exercise and could be an effective exercise strategy as well.

## Introduction

Stroke is the third leading cause of mortality and disability worldwide (Xiang et al., 2019; Turon et al., 2020). Despite growing approaches to treatment, many patients are left with a lifelong disability. Various conditioning strategies play important roles in reducing ischemic and reperfusion injury from stroke (Serviddio et al., 2008; Lemoine et al., 2017; Wang J. L. et al., 2020) to improve the prognosis and quality of life in stroke victims. Ischemic postconditioning is one of the strategies widely used to attenuate stroke-induced neural damage in animals and humans (Landman et al., 2019). In this rehabilitative method, cycles of transient ischemia to the distal limbs after a vascular accident, such as a stroke, stimulate physiologic responses that confer protection to the ischemic brain (Zhao J. J. et al., 2018). Alternatively, exercise conditioning is believed to be an effective strategy that attenuates the detrimental effects of ischemia and enhances various cognitive abilities, such as motor function (Thijssen et al., 2018). More specifically, prophylactic exercise, known as exercise preconditioning, has been widely reported to confer rehabilitative benefits in the post-stroke brain, such as the reduction of brain infarct volume and the promotion of angiogenesis, synaptogenesis, and neurogenesis after ischemia (Hafez et al., 2019; Lee et al., 2019; Terashi et al., 2019). Similarly, the benefits of post-ischemic exercise intervention were initially established in the context of myocardial injury in animal models. It has since been established that exercise postconditioning exerts beneficial effects in improving cardiovascular outcomes after myocardial ischemia (Szabo et al., 2019; Lee et al., 2020). However, the short-term effect of exercise postconditioning on the outcome of ischemic stroke has not been established. A growing body of evidence has demonstrated that higher intensity post-cerebral ischemia exercise yields beneficial effects in reducing brain infarct size, is an effective strategy for ameliorating physical disability, and leads to favorable outcomes after 3 months, which suggest that this exercise strategy has potential neuroprotective effects after stroke (Boyne et al., 2016; Luo et al., 2019; Andrews et al., 2020). Other studies suggest that mild exercise after stroke is superior in promoting recovery and improvement from brain damage (Zhu et al., 2015; Nie et al., 2016). The present conflicting conclusion elucidates the vital principle that exercise postconditioning intensity is an important determinant of neurological outcome after a stroke that merits close investigation. Patients may differ in their abilities and may be more vulnerable to harm from exercise, and thus prescribing the appropriately intense exercise and minimizing superfluous therapy is vital to their safe and successful rehabilitation. The appropriate intensities of exercise postconditioning and an understanding of its associated mechanisms need to be explored, as it may help direct the clinical application of exercise-based neuroprotection.

Many recent studies have demonstrated that cerebral ischemic injury is correlated to stress in the endoplasmic reticulum (ER), a multifunctional organelle responsible for protein synthesis and processing (Gong et al., 2017). Certain stressors, such as ischemia, disrupt homeostasis and cause ER stress, which triggers the unfolded protein response to minimize levels of unfolded proteins and to maximize cell viability (Ahsan et al., 2019). Prolonged environmental stress triggers signal transduction pathways that lead to apoptosis and thereby heighten pathological processes, leading to cerebral damage (Chi et al., 2019). Endoplasmic reticulum stress exerts its vital role in stroke-induced neural apoptosis (Mohammed Thangameeran et al., 2020) through activation of downstream CCAATenhancer-binding protein homologous protein (CHOP) and caspase-12 (Chi et al., 2019; Chu et al., 2019; Li Y. et al., 2020). SIRT1 is a nicotine adenine dinucleotide (NAD+)-dependent enzyme that deacetylates numerous transcription factors in response to ischemic stress and is involved in various biological pathways (Chen et al., 2005). Notably, it suppresses ER stress signals through the eEF2K/eEF2 pathway (Pires Da Silva et al., 2020) by increasing the expression of protective molecular chaperones (Wang F. et al., 2020) and by decreasing ROS production, which are potent ER stress agonists (Park et al., 2020). Its expression has also been shown to be elevated by post-stroke exercise, which suggests that exercise protects against ischemic damage by limiting ER stress (El Hayek et al., 2019; Jia et al., 2019). However, the precise mechanism by which exercise postconditioning affects the SIRT1/ER stress signal after stroke remains undetermined. The relationship between various exercise intensities and SIRT1/ER stress levels may help elucidate the mechanism of exercise-induced neuroprotection after ischemic stroke and guide efforts to optimize exercise intensity in postconditioning after stroke. We used an ischemia/reperfusion rat model to define the effect of exercise postconditioning after stroke and determine the relationship between SIRT1 and ROS/ER stress. Although the present study did not determine this relation, as the first step, we intended to assess the expression of SIRT1 and ER stress proteins following ischemia/reperfusion injury. These results might suggest a potential association of these molecules and provide a base for our future study regarding the regulation of SIRT1 on the ER stress pathway. The results of this study suggest a pathway for neuroprotection mediated by exercise postconditioning after stroke.

## Materials and Methods

### Animals

**Figure 1:**
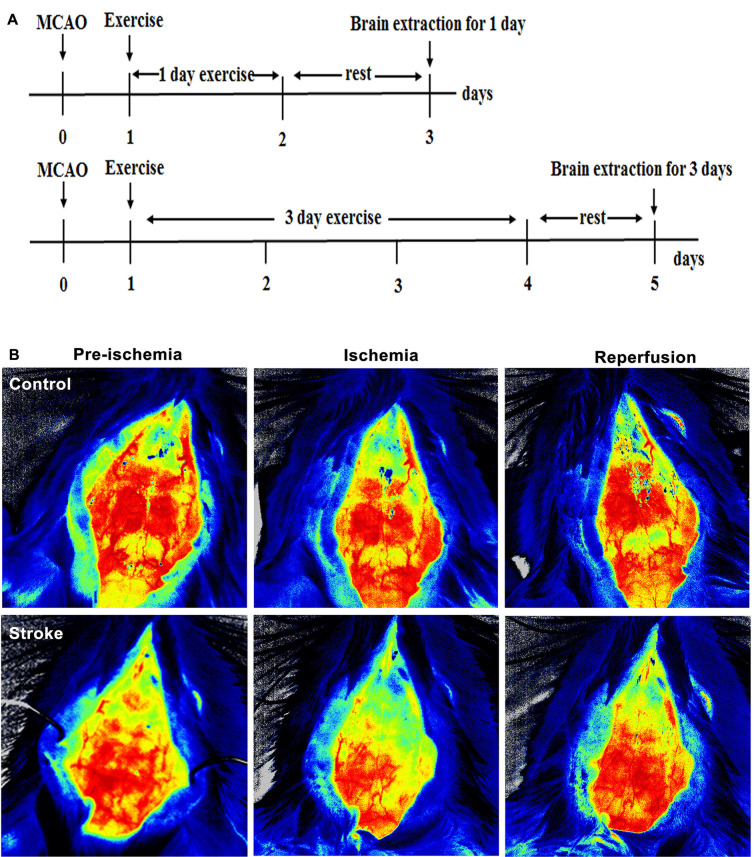

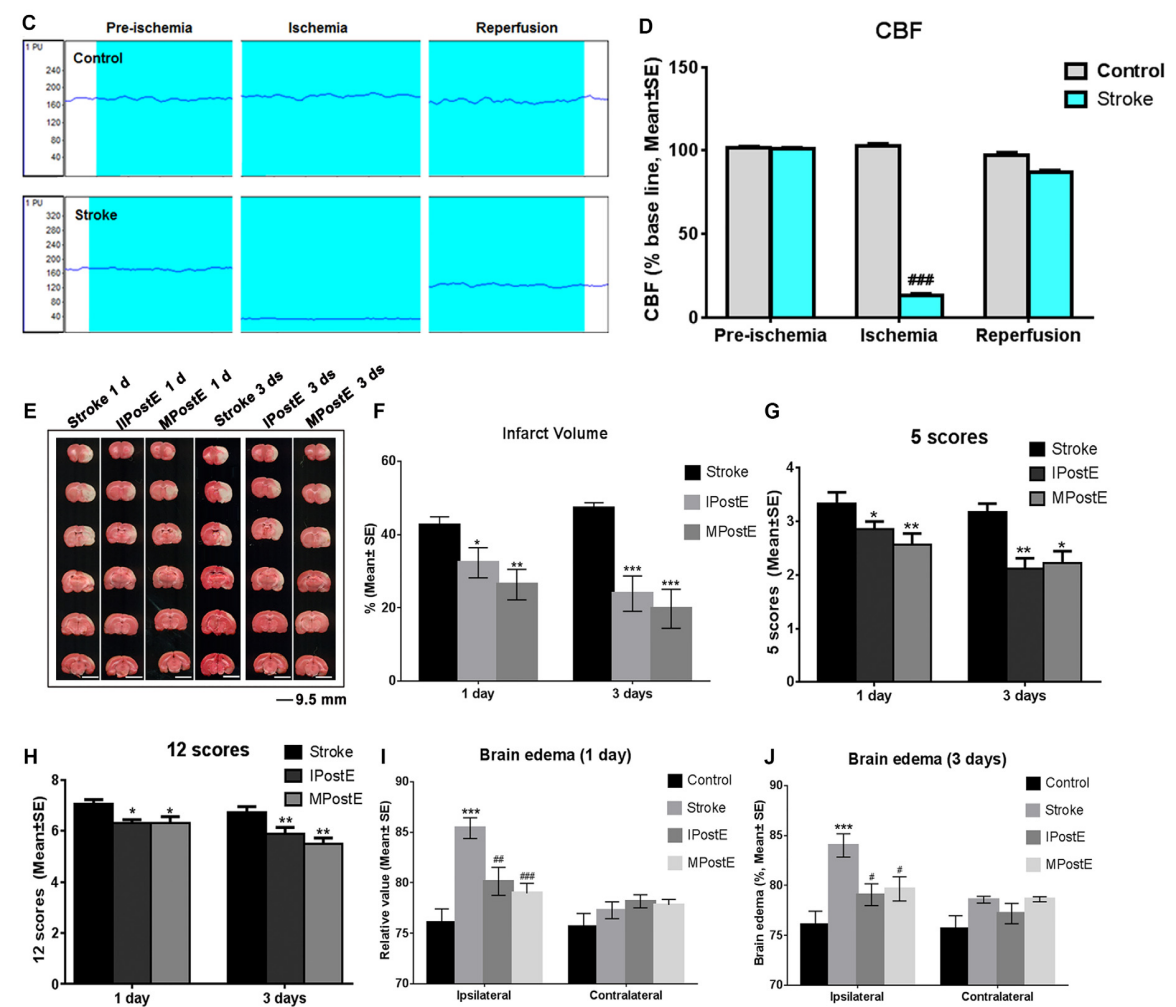
Brain infarct and neurological deficits. **(A)** Illustration of the experimental timelines. Rats were subjected to 2 h middle cerebral artery occlusion (MCAO), followed by treadmill exercise 1 day after reperfusion for up to 2 or 4 days. **(B–D)** Representative images of monitored cerebral blood flow (CBF). **(E)** 2,3,5-triphenyltetrazolium chloride (TTC) histology demonstrating exercise postconditioning (PostE)-induced infarct volume reduction in the penumbra region of the ischemic territory supplied by the middle cerebral artery. **(F)** Quantification of infarct volume reduction by exercise conditioning. Compared to the stroke group, brain infarct (*n* = 7 respectively, *p < 0.05, 1 day; ***p < 0.001, 3 days) significantly decreased in both intense and mild PostE groups. Neurological deficits after both PostE in 2 h MCAO, measured using the 5 score **(G)** and 12 score systems **(H)**, were decreased significantly (*p < 0.05) after either mild or intense PostE. **(I–J)** PostE attenuated ipsilateral stroke-induced brain edema significantly vs. ipsilateral stroke group without exercise (^#^*P* < 0.05) at day 1 **(I)** and days 3 **(J)**. *, **, and *** represent MPostE or IPostE vs. stroke; ^#^ , ^##^ , and ^###^ represent IPostE vs. MPostE.

A total of 175 adult male Sprague–Dawley rats (280–300 g, Vital River Laboratory Animal Technology Company, Limited, Beijing, China) were used in this study. The protocol, following the NIH Guide for the Care and Use of Laboratory Animals, was approved by the Animal Care and Use Committee of Capital Medical University (20180901). Animals were randomly divided into four groups: control (*n* = 7), middle cerebral artery occlusion (MCAO) without exercise (*n* = 56), MCAO with IPostE (*n* = 56), and MCAO with MPostE (*n* = 56). Both PostE protocols were initiated after 24 h of reperfusion and select animals from every group were sacrificed on days 1 and 3 after exercise for further biochemical analysis, as shown in Figure 1A. Seven rats from each group were used for the histological assay, Western Blotting, ROS detection, or brain edema analysis.

### Focal Cerebral Ischemia and Reperfusion

The animals were subjected to transient right MCA occlusion according to the method we described previously (Chen et al., 2020). Briefly, rats were anesthetized in a chamber using 3% isoflurane and a mixture of 70% nitrous oxide and 30% oxygen. The rats were then transferred to a surgical table, where anesthesia was maintained with a facemask that delivered 1% isoflurane from a calibrated precision vaporizer. Poly-L-lysine-coated nylon (4.0) sutures were used to generate infarcts with minimal inter-animal variability. During the unilateral, two-hour MCAO procedure, cerebral blood flow (CBF) and rectal temperature were monitored continuously. CBF was monitored using the laser speckle technique according to the manufacturer’s instructions. CBF before MCAO was defined as the baseline. Quantitative measurement of CBF 2 min before, immediately after MCAO, and at reperfusion were recorded as a percentage of baseline levels. Rectal temperatures were maintained between 36.5 and 37.5°C using a heating pad and a heating lamp. Ipsilateral ischemic hemispheres were used for further molecular analysis.

### Exercise Postconditioning

All ischemic animals were randomly assigned to intense exercise, mild exercise, or non-exercise control groups. Exercise animals were run on a four-lane treadmill (ZS-PT-II, ZS Dichuang Instruments, Inc., Beijing, China), at a constant speed of 30 m/min for 30 min each day for the IPostE; or at 12 m/min for 30 min each day for the MPostE (Li F. et al., 2020). The rats were well enough for the exercise protocols at 24 h of spontaneous recovery, with recovery comparable to the performances on the treadmill at 3 days. For pre-conditioning to exercise, rats were required to perform treadmill training at a constant speed (10 m/min) for 10 min/day for 3 days before MCA occlusion. Although typical motor function impairment (movement in a circle) was observed, the animals moved around in the cage and were able to run on the treadmill for 30 min. Our previous studies, as well as others, have performed the exercise programs 24 h after MCAO, in which most animals were able to run continuously for 30 min (Li et al., 2017a,b,c; Pin-Barre et al., 2017; Li F. et al., 2020). From the beginning of group assignments until 1 or 3 days thereafter, all animals were housed in groups of threes in standard cages for equal times.

### Cerebral Infarct Volume

After one or 3 days of exercise or non-exercise, the rats’ brains were dissected, cut into 2-mm-thick slices with a brain matrix, and treated with 2,3,5-triphenyltetrazolium chloride (TTC, Sigma, USA) for staining. An indirect method for calculating infarct volume was used to minimize error caused by edema, as described previously by us (Liu et al., 2020).

### Neurological Deficit

The modified scoring systems (5 and 12 scores) proposed by Zea Longa (5 scores) and Belayev et al (12 scores) were used to examine the severity of neurological deficits in rats before and after 24 h reperfusion (Li et al., 2019a). After MCAO, the modified scoring systems for neurological deficits were used to confirm brain injury. If the scores were 1 or below, the MCAO was considered unsuccessful and the rats were excluded (19, about 10%) from further studies. The exclusion was confirmed by an autopsy that lacked a core.

### Brain Edema

Brain edema was evaluated through measurements of tissue water content to demonstrate brain swelling, as described previously by us (Li et al., 2019b). Following animal sacrifice, separated right and left hemispheres were immediately weighed to obtain wet weight (WW). The tissue was then dried in an oven at 70°C for 72 h and weighed again to obtain the dry weight (DW). The formula (WW − DW)/WW × 100% was used to calculate the water content and is expressed here as a percentage of WW.

### Apoptotic Cell Death

For the quantification of apoptosis-related DNA fragmentation, a commercial enzyme immunoassay was used to determine the cytoplasmic histone-associated DNA fragments (Cell Death Detection ELISA; MLBIO, Shanghai, China). The degree of apoptosis was quantified by the amount of cytoplasmic histone-associated DNA fragments in the stroke and variable groups at 1 or 3 days after PostE.

### ROS Assay

As described previously by us (Chen et al., 2018), an adult brain dissociation kit (Miltenyi Biotec, Bergisch Gladbach, Germany) was used for brain cell isolation. Cell pellets were stained with ROS probes (S0063, Dihydroethidium, Biyuntian, Shanghai, China) and analyzed on a FACS Calibur flow cytometer with the Cell Quest software (BD, San Jose, CA, USA).

### Protein Expression

After 1 or 3 days of exercise or non-exercise, rats were sacrificed for Western blot analysis. Tissue samples from the ipsilesional ischemic hemispheres were harvested and processed as described previously by us (Li et al., 2019a). Briefly, samples were incubated with a primary antibody rabbit anti-BAX (1:1,000, ab32503, Abcam, MA, USA), rabbit anti-Bcl-2 (1:500, ab196495, Abcam), rabbit anti-caspase-3 (1:1,000, ab13847, Abcam), rabbit anti-IRE1 (1:1,000, 14C10, CST, MA, USA), rabbit anti-GRP78 (1:1,000, 3183S, CST), rabbit anti-PERK (1:1,000, 27528-1-AP, PROTEINTECH, IL, USA), rabbit anti-ATF6 (1:1,000, ab203119, Abcam), rabbit anti-caspase-12 (1:1,000, ab62484, Abcam) and rabbit anti-CHOP (1:1,000, 2895S, CST) for 24 h at 4°C. The samples were further incubated with goat anti-rabbit IgG-HRP secondary antibody (1:1,000, sc-2004, Santa Cruz, Dallas, TX, USA) for all primary antibodies. Western blot images for molecules were analyzed using an image analysis program (ImageJ 1.42, National Institutes of Health, Bethesda, MD, USA) to quantify protein expressions according to relative image density. The calculations of Western blotting images were normalized according to their corresponding β-actin.

### Statistical Analysis

Statistical analyses were performed with SPSS Statistics for Windows, Version 17.0 (SPSS Inc., Chicago, IL, USA). Differences among groups were assessed using one-way ANOVA with significance levels of *p* < 0.05. *Post hoc* comparisons among groups were performed using the least significant difference method.

## Results

### Cerebral Blood Flow (CBF)

Compared to CBF before ischemia, a significant decrease was observed after ischemia (Figures 1B–D).

### Brain Infarction and Neurological Defects

Stroke-induced brain infarction and the infarct volumes were decreased significantly (*p < 0.05) in both PostE groups at 1 and 3 days (*n* = 7 respectively, Figures 1E,F). Infarct volumes observed in the stroke groups were 42.5% at 1 day and 47.2% at 3 days (*n* = 7 respectively, Figures 1E,F). Infarction was significantly decreased by both exercise protocols, with IPostE decreasing to 32.3% and 19.7% on days 1 and 3, respectively (**p < 0.01), and MPostE decreasing to 26.3% and 24.8% on days 1 and 3, respectively (*p < 0.05). Neurological deficits detected by the 5- (*n* = 7, Figure 1G) and 12-point (*n* = 7, Figure 1H) score systems were 3.3 and 7.1 points (1 day), then 3.2 and 6.7 points (3 days) in the stroke groups. The 5-point score was decreased significantly after MPostE (**p < 0.01, 2.6 points at 1 day; *p < 0.05, 2.2 points at 3 days) and IPostE (*p < 0.05, 2.9 points at 1 day; **p < 0.01, 2.1 points at 3 days). The same trends were also seen in the 12-point score.

### Brain Edema

On the ipsilateral side, a significant (***p < 0.001) increase in water content was observed in ischemic rats (85.4% at 1 day and 84.0% at 3 days) as compared to control rats (76.1%) (*n* = 7 respectively, Figures 1I,J). Brain edema was significantly decreased by both exercise protocols, with IPostE decreasing to 80.1% and 79.1% on day 1 and 3, respectively (*p < 0.05), and MPostE decreasing to 79.0% and 79.7% on days 1 and 3, respectively (*p < 0.05; *n* = 7 respectively, Figures 1I,J). PostE made no significant difference to the water levels when compared with the control group in the contralateral hemisphere of ischemic rats.

### Apoptosis and Apoptotic Proteins

**Figure 2:**
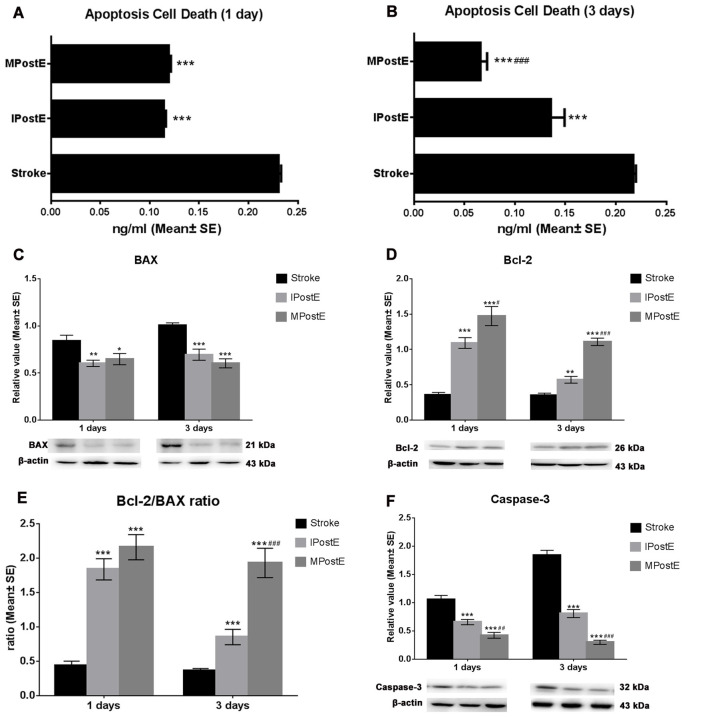
Apoptosis and apoptotic proteins. **(A,B)** Cell death reduction due to PostE at 1 and 3 days, represented by apoptotic protein levels. Apoptotic cell death was detected at 1 and 3 days; both mild and intense PostE significantly (*n* = 7 respectively, ***p < 0.001) decreased cell death (Figures 1H,I), and a further significant decrease was noted (^###^*p* < 0.001) in the mild exercise group. **(C–F)** Representative images of BAX, Bcl-2, and Caspase-3 detected by Western Blot. Both intense (**p < 0.01) and mild (*p < 0.05) PostE (*n* = 7 respectively) decreased expressions of BAX **(C)** and Caspase-3 **(F)** at 1 and 3 days; significantly decreased Caspase-3 expression was seen with mild PostE (^###^*p* < 0.001). Compared to the stroke group, both mild and intense PostE significantly increased protein expression of Bcl-2 and Bcl-2/BAX ratio at 1 and 3 days **(D,E)**. Levels of Bcl-2 (**p < 0.01; **D**) and Bcl-2/BAX ratio (***p < 0.001; **E**) were found to be increased in IPostE rats at 1 and 3 days; similar results were seen with MPostE. ^#^ and ^##^ represent IPostE vs. MPostE.

Apoptotic cell death was detected at 1 and 3 days (*n* = 7 respectively, Figures 2A,B). Both mild and intense exercise significantly decreased cell death (***p < 0.001), with further significant decrease observed in the mild exercise group at 3 days (^###^*p* < 0.001; Figure 2B). Levels of BAX, Bcl-2, and Caspase-3 were detected by Western blot (*n* = 7 respectively, Figures 2C–F). Compared to the stroke group, MPostE significantly decreased pro-apoptotic protein expressions, such as BAX (*p < 0.05 at 1 day and ***p < 0.001 at 3 days, Figure 2C) and Caspase-3 (***p < 0.001 at 1 day and ***p < 0.001 at 3 days, Figure 2F), and increased anti-apoptotic protein expression, such as Bcl-2 (***p < 0.001 at 1 day and ***p < 0.001, 1.1 at 3 days, Figure 2D). The same trends were also seen in the IPostE group. Additionally, we found that MPostE further significantly increased Bcl-2 level (^#^*p* < 0.05, 1 day; ^###^*p* < 0.001, 3 days, Figure 2D) and decreased Caspase-3 level (^##^*p* < 0.01, 1 day; ^###^*p* < 0.001, 3 days, Figure 2F) as compared to IPostE group. Moreover, the Bcl-2/BAX ratio in the stroke groups was 0.4 at both 1 and 3 days but significantly increased in both IPostE (***p < 0.001, 1.8 at 1 day and 0.9 at 3 days) and MPostE groups (***p < 0.001, 2.2 at 1 day and 1.9 at 3 days, Figure 2E). A further significant Bcl-2/BAX ratio increase was noted in the MPostE group at 3 days compared to the IPostE group (^###^*p* < 0.001, Figure 2E).

### Expression of ER Stress

**Figure 3:**
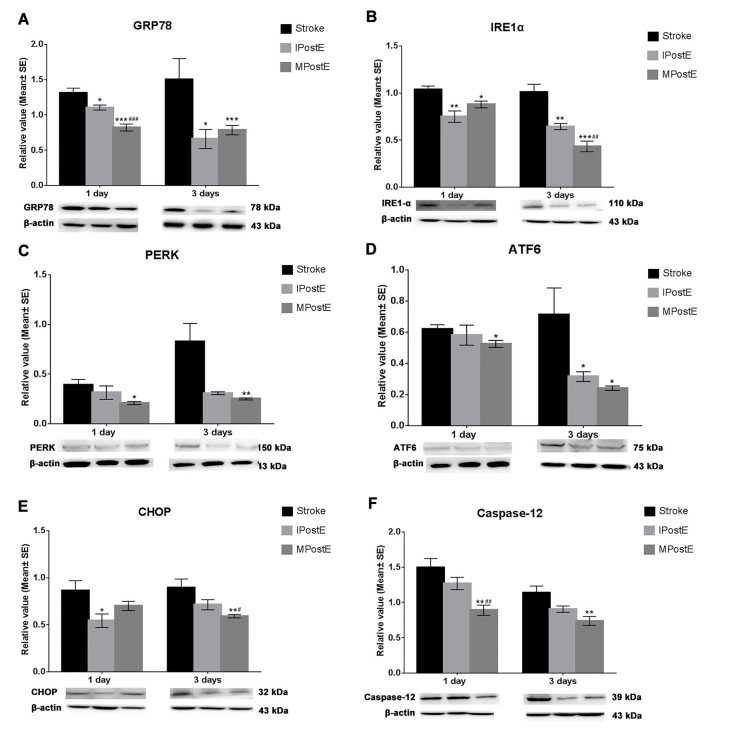
Expression of endoplasmic reticulum (ER) stress. Both mild and intense PostE yielded a significant decrease in ER stress protein levels at 1 and 3 days. Compared to the stroke group, levels of **(A)** GRP78 (*p < 0.05, 1 and 3 days), **(B)** IRE1α (**p < 0.01, 1 and 3 days), **(C)** PERK, **(D)** ATF6 (*p < 0.05, 3 days), **(E)** CHOP (*p < 0.05, 1 day) and **(F)** Caspase-12 were significantly decreased in IPostE groups (*n* = 7 respectively). Similar results were also seen in the MPostE group. The protein levels were further decreased in the MPostE group compared to the IPostE group. The significance of the decreases were as follows: **(A)** GRP78 (^###^*p* < 0.001, 1 day), **(B)** IRE1α (^##^*p* < 0.01, 3 days), **(E)** CHOP (*p < 0.05, 3 days) and **(F)** Caspase-12 (*p < 0.05, 1 day). *** represents MPostE or IPostE vs. stroke; ^#^ represents IPostE vs. MPostE.

The levels of ER stress proteins in the stroke group were increased, including GRP78 (1.3, 1 day; 1.5, 3 days, Figure 3A), IRE1α (1.0, 1 and 3 days, Figure 3B), PERK (0.4, 1 day; 0.8, 3 days, Figure 3C), ATF6 (0.6, 1 day; 0.7, 3 days, Figure 3D), CHOP (0.7, 1 day; 0.9, 3 days, Figure 3E) and Caspase-12 (1.5, 1 day; 1.1, 3 days, Figure 3F). As compared to the stroke group, MPostE and IPostE significantly decreased expressions of GRP78 (Figure 3A), IRE1α (Figure 3B), PERK (Figure 3C), ATF6 (Figure 3D), CHOP (Figure 3E), and Caspase-12 (Figure 3F) (*n* = 7 respectively). MPostE was shown to significantly further reduce the levels of ER stress proteins compared to IPostE. These results demonstrate that the ER stress pathway may be involved in the regulation of apoptosis induced by ischemia/reperfusion and that varying degrees of exercise intensity may preferentially affect this pathway.

### ROS Production and SIRT1 Expression

**Figure 4:**
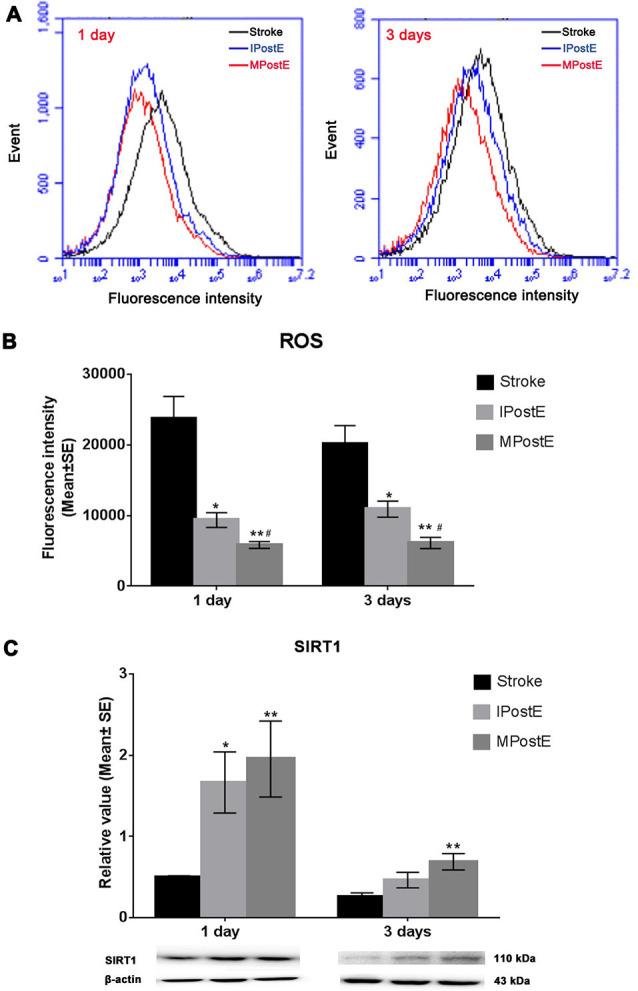
ROS production and SIRT1 expression. **(A,B)** Flow cytometry assay demonstrated that both mild and intense PostE groups observed decreased ROS production at 1 and 3 days (*n* = 7 respectively, *p < 0.05); between the two groups, MPostE further significantly decreased ROS production (*p < 0.05). **(C)** Compared to the stroke group, levels of SIRT1 in MPostE groups were significantly increased (*n* = 7 respectively, *p < 0.05, 1 day; **p < 0.01, 3 days). Similar results were also seen with IPostE. ^#^represents IPostE vs. MPostE.

ROS production levels, measured by flow cytometry assay, were significantly decreased by exercise when compared to the levels raised after stroke (*p < 0.05; *n* = 7 respectively, Figures 4A,B). The fluorescence intensity of ROS level in the stroke group was 23, 733.1 at 1 day and 20, 132.9 at 3 days (Figure 4B). Compared to the stroke group, MPostE (**p < 0.01, 5, 856.8 at 1 day and 6, 131.9 at 3 days, Figure 4B) and IPostE (*p < 0.05, 9, 376.6 at 1 day and 10, 920.1 at 3 days, Figure 4B) significantly decreased ROS production. MPostE further significantly decreased ROS production (^#^*p* < 0.05) at 1 and 3 days compared to IPostE. Furthermore, both PostE enhanced SIRT1 expressions in ischemic rats (Figure 4C). Protein expression of SIRT1 was significantly enhanced by MPostE (**p < 0.01, 2.0 at 1 day and 0.7 at 3 days, Figure 4C) and IPostE (*p < 0.05, 1.7 at 1 day, Figure 4C; *n* = 7 respectively). The present data indicate that SIRT1 and ROS may be involved in the ER stress pathway and exercise postconditioning.

## Discussion

In this study, we demonstrated that recovery from ischemic brain damage is enhanced by exercise postconditioning after stroke. Our results show that both mild and intense exercise regimes performed after stroke reduce brain edema and infarct size, neurological deficits, ROS production, and apoptotic cell death. Mild exercise induced a greater degree of apoptosis prevention in comparison to intense exercise. Reduced brain injury was associated with an increase of anti-apoptotic proteins (Bcl-2) and Bcl-2/BAX ratio, and a decrease of pro-apoptotic proteins (BAX and caspase-3) and ER stress (GRP78, IRE1, PERK, ATF6, Caspase-12, CHOP). Furthermore, post-stroke exercise increased SIRT1 expression and had an inverse relationship with ROS levels.

Ischemic conditioning, which includes pre-and post-conditioning, is a nonpharmacological approach to inducing neuroprotection that is performed by the induction of intermittent blood flow restriction to non-vital organs. It has been widely reported to ameliorate brain and myocardial injury after an ischemic event (Guo et al., 2019). It’s characterization of periodic ischemic induction is similar to the periodically scheduled exercise procedure that is also used as a form of cardioprotective conditioning (Thijssen et al., 2018). Chronic remote ischemic conditioning and regular exercise may share similar underlying cellular protective mechanisms such as increased antioxidant capacity, inhibition of autophagy, and the involvement of the NO pathway and inflammatory system (Zhao W. et al., 2018). Indeed, both exercise and ischemic conditioning are likely to employ similar mechanisms to enhance endogenous angiogenesis, synaptogenesis, and neurogenesis to improve recovery from ischemic injury. In light of these parallels, it is likely that regular post-stroke exercise conditioning, just like ischemic conditioning, has widespread health benefits and the potential for enhancing rehabilitation from cerebrovascular events. Though initially described through the scope of cardioprotection, there is growing literature substantiating the beneficial effects of exercise conditioning in restoring brain function after ischemic injury (Otsuka et al., 2016; Sakakima, 2019). Prophylactic exercise such as pre-ischemic exercise has been studied in stroke medicine and has been found in our previous studies to exert neuroprotective effects by improving recovery from blood-brain barrier (BBB) dysfunction and reducing brain infarct size (Davis et al., 2007; Otsuka et al., 2019). The present study’s results further suggest that post-ischemic exercise conditioning, similar to pre-ischemic exercise conditioning, is also capable of inducing neuroprotective effects.

Previous studies have demonstrated that post-myocardial injury exercise exerted beneficial postconditioning effects by decreasing inflammatory reactions and ameliorating antioxidative statuses (Szabo et al., 2019), and also played a beneficial role in reducing liver carcinogenesis (Aguiar e Silva et al., 2012). A recent study indicated that exercise postconditioning after doxorubicin suppressed doxorubicin-induced cardiotoxicity (Lee et al., 2020). Another indicated the involvement of the BDNF/TrkB pathway in the increased myocardial angiogenesis and improved cardiac function seen in rats the exercised after myocardial infarction (Wang et al., 2018). These pieces of evidence imply the role of exercise postconditioning as a beneficial post-stroke intervention. Our previous studies support these findings and this notion as they demonstrated that exercise improved metabolism and decreased neuroinflammation at 1 and 3 days after stroke (Li et al., 2017a; Li F. et al., 2020). Although a rota-rod exercise style was used, both early intense and mild exercise conferred the similar neuroprotective effect by minimizing brain damage and apoptosis (Li et al., 2017c). The present results were further supported by the results of other groups, which indicated that various types and intensities of early exercise postconditioning accelerated CBF (Pianta et al., 2019), decreased infarct volume (Tian et al., 2013; Pan et al., 2020) and improved functional outcomes (Pianta et al., 2019). However, a few studies claimed that no obvious improvements were found after post-stroke exercise treatment on brain infarct and recovery (Matsuda et al., 2011; Cui et al., 2020). These studies employed models of 90-min ischemia or permanent MCAO models in their exercise procedures, whereas we used a model of 120 min temporary ischemia and induced a relatively small size of infarction. These differences may explain the lack of significant differences observed after exercise. Also, the relatively low intensity of the rota-rod training model or insufficient exercise duration used in their exercise procedures may contribute to the discrepancy of these conclusions with our present results. This perspective was also supported by our previous studies, in which the early high-intensity rota-rod exercise exacerbated brain damage in the short-term. We demonstrated that early post-stroke exercise enhanced apoptosis through the expression of proapoptotic proteins (Li et al., 2017c) and aggravated brain damage through hyperglycolysis and NOX activation (Shen et al., 2016). Thus, the apparent inconsistencies in the effect of exercise training on brain infarct and recovery may due to the discrepancies in the duration, timing, or style of post-stroke exercise (Li et al., 2017a). Future studies are necessary to fully elucidate the effect of post-stroke exercise on brain injury and the optimal parameters for this proposed therapy. Additionally, future studies are needed to examine the impact of varying exercise intensities on other markers and participants of ER stress, such as molecular chaperones (e.g., GRP78) and caspases (e.g., caspase-9 and 12).

Previous studies have also found that the neurological outcomes of post-stroke exercise differed according to the exercise regimen intensities (Bell et al., 2015; Xing et al., 2018). Of these, some claimed that higher intensity exercise post-stroke yielded better functional recovery (Luo et al., 2019; Andrews et al., 2020), while others suggested that mild exercise resulted in superior neuroprotection (Lee et al., 2009; Shih et al., 2013), citing that exercise that is too intense may cause spikes in endogenous corticosteroid levels that limit post-stroke neurogenesis (Yagita et al., 2006). In no strong agreement with either of these findings, the present results indicated similar neuroprotective effects of both mild and intense doses of exercise postconditioning, although mild exercise did periodically have greater protection against apoptotic cell death. The protocol with two doses utilized in the present study was partially based on the previous studies on exercise intensity from us (Curry et al., 2010; Li F. et al., 2020) and others (Zhang et al., 2012; Gronwald et al., 2019). Following the principle that physical exercise regimens are reproducible (Gronwald et al., 2019), we set the highest achievable exercise training intensity to be the speed at which the rats could no longer run due to fatigue within 3 min of exercise onset. The therapeutic doses of physical exercise training used in our study were calculated as 40% of this maximum velocity for mild exercise training, which amounted to approximately 15 m/min, and 80% of the maximum velocity for intense exercise training, which was about 32 m/min (Zhang et al., 2012; Li F. et al., 2020). To further accentuate the difference between the two categories, we reduced the mild exercise group’s speed to a maximum of 12 m/min as done by previous studies (Tian et al., 2013; Zhang et al., 2017; Tang et al., 2018; Li F. et al., 2020). For the high-intensity group, we selected 30 m/min because we have employed this speed in our previous work, in which we found that it reduced brain damage (Ding et al., 2006), blood-brain barrier dysfunction (Guo et al., 2008), and brain inflammation in stroke (Curry et al., 2010). Another key finding from our previous work was the influence of initiation time on post-stroke rehabilitation outcomes: initiation of exercise 6 h post-stroke exacerbated brain damage, while exercise deferment for 1–3 days avoided exacerbation of brain damage (Li et al., 2017a, Li F. et al., 2020). Therefore, an initiation time of 24 h after stroke was selected for this study.

In this study, we aimed to observe the short-term neuroprotective effect of post-stroke exercise conditioning on the infarcted brain. Previous studies have indicated that post-stroke days 1–3 are critical timepoints at which exercise both decreases brain damage and improves functional outcomes. Indeed, this time range avoids the brain damage exacerbation seen in very early exercise interventions while preserving the benefit of prompt post-stroke rehabilitation (Li et al., 2017a). Also, in our recent reports (Li F. et al., 2020), we have compared the long-term effects of these two exercise procedures for up to 28 days, which is the commonly used time point for long-term outcomes. In our future study, we may explore the long-term effect of the exercise protocol by expanding it to 60 days after focal cerebral ischemia to investigate the benefits of expanding the therapeutic timeframe. This will allow us to determine whether postconditioning can also impact the remodeling processes that are crucial to long-term recovery from a cerebrovascular event.

The alterations in apoptotic death protein levels observed in the present study aligned with the current understanding of brain injury reduction, which is accomplished by time-sensitive, early suppression of neural apoptosis in the penumbra during the early stages of ischemia (Uzdensky, 2020). Previous studies have reported that ischemic or exercise conditioning suppresses neural apoptosis by regulating the expression of pro-and anti-apoptotic proteins (Zhou et al., 2011; Terashi et al., 2019). Our previous studies have indicated that early transient post-stroke exercise also reduces brain infarct in the penumbra by mediating the expression of pro-and anti-apoptotic proteins to favor functional recovery (Li et al., 2017a,c). Upregulation of anti-apoptotic proteins such as Bcl-2 in conjunction with an increased Bcl-2/BAX ratio appears to play a key role in reducing damage from ischemic injury and enabling rehabilitation (Ruan et al., 2020). Bcl-2 serves as a regulator of apoptosis-regulatory proteins such as caspase-3, which is an aspartate-specific cysteine protease that serves as an important executioner in cell death (Yang et al., 2019). Exercise preconditioning has been reported to reduce brain damage and neuronal apoptosis through enhancing the Bcl-2/Bax ratio and suppressing caspase-3 after focal brain ischemia in rats (Otsuka et al., 2019; Terashi et al., 2019). A series of studies have also shown that ischemic pre- or postconditioning alleviated ischemia/reperfusion injury by suppressing BAX-mediated apoptosis (Zhou et al., 2011; Zhang et al., 2018). Hence, the concurrent enhancement of Bcl-2 and suppression of proapoptotic factors such as caspase-3 and BAX may be contributing to the decrease of neuronal cell death following ischemic stroke. Therapies that catalyze the restorative mechanisms and suppress the apoptotic processes are thereby likely to encourage recovery from ischemic stroke and minimize acquired disability.

ER stress is one of the complex sets of mechanisms involved in apoptosis (Zheng et al., 2018). Activation of ER stress is stimulated by the upregulation of GRP78, which subsequently induces apoptosis signaling mediated by CHOP and caspase-12 through IRE1α-XBP-1, PERK-ATF4, and ATF6-dependent pathways (Zhang and Kaufman, 2008). Caspase-12 regulates ER stress-induced apoptosis signaling by activating caspase-9, which consequently activates caspase-3 (Datta et al., 2018; Rong et al., 2020). CHOP, a transcription factor, activates the expression of anti-apoptotic and pro-apoptotic proteins including those of the Bcl-2 family (Coker-Gurkan et al., 2019). Previous studies showed that ischemic postconditioning ameliorated cerebral ischemic injury by favoring these ER-stress-mediated anti-apoptotic, rather than pro-apoptotic mechanisms (Mahfoudh-Boussaid et al., 2012; Liu et al., 2014). Exercise preconditioning also exerted a cardioprotective effect *via* alleviating apoptosis regulated by ER stress (Korzeniowska Kubacka, 2011). These results are as per the present study, which showed that exercise post-conditioning mediated a decrease in the ER stress pathway, suggesting a potential anti-apoptotic mechanism of neuroprotection after stroke.

The cause-effect relationship between ROS and ER stress signaling has been well studied (Cao et al., 2018; Park et al., 2020); ROS was reported to be a potent trigger of ER stress and to subsequently induce apoptosis in cerebral ischemic stroke (Shi et al., 2019; Wei et al., 2019). SIRT1 was reported to suppress ROS release (Park et al., 2020), which protects the heart from ER stress-induced apoptosis (Pires Da Silva et al., 2020). The protective effects of ischemic postconditioning and post-myocardial infarction exercise were accomplished by enhancing SIRT1 expression (Ahsan et al., 2019; Ding et al., 2019), indicating its potential regulative role in protective post-stroke exercise. These previous studies and the strong inverse correlation of SIRT1 and ER stress protein expression observed in the present study suggests an association of SIRT1 and ER stress pathway in exercise postconditioning-induced neuroprotection. The link of SIRT1 to ER stress molecules was relatively weak in the present study. Our further investigation will additionally address the cause-and-effect link between SIRT1 and ER stress pathways by using both SIRT1 gene knockout animals and a SIRT1 inhibitor. This will enable us to examine the extent of the impact of SIRT1 on ER stress. Also, the interaction of these molecules will be determined, which will clarify the mechanisms that facilitate the ER stress pathway. Although we did not explicitly study the regulation of SIRT1 on the ER stress pathway, our results suggest a potential link between the molecules. Our findings could be a basis to further clarify the participation of SIRT1 in ER stress in stroke.

In conclusion, this study confirmed the beneficial effect of exercise postconditioning with either intense or mild doses after stroke. Our results further demonstrated that intense exercise postconditioning did not confer superior benefits to its milder counterpart, suggesting that an easier procedure with mild exercise conditions would be an appropriate protocol for stroke rehabilitation procedure. Moreover, the results may provide a base for our future study regarding the regulation of SIRT1 on the ER stress pathway in the biochemical processes underlying exercise postconditioning-induced neuroprotection.

## Data Availability Statement

The raw data supporting the conclusions of this article will be made available by the authors, without undue reservation.

## Ethics Statement

The animal study was reviewed and approved by the Animal Care and Use Committee of the Capital Medical University.

## Author Contributions

FL conducted the animal and biochemical experiments employed in this research. FL, XG, HL, and MW were instrumental in preparing and revising the manuscript. YD was responsible for the experimental design, manuscript preparation, and revision. All authors contributed to the article and approved the submitted version.

## Conflict of Interest

The authors declare that the research was conducted in the absence of any commercial or financial relationships that could be construed as a potential conflict of interest.
