# Supplementary material for: Neuroprotective Effects of Exercise Postconditioning After Stroke via SIRT1-Mediated Suppression of Endoplasmic Reticulum (ER) Stress
Source: Front Cell Neurosci. 2021 Feb 16;15:598230. doi: 10.3389/fncel.2021.598230 (PMC7920953; doi:10.3389/fncel.2021.598230)
Supplement: Supplementary file 1 [file Data_Sheet_1.PDF]

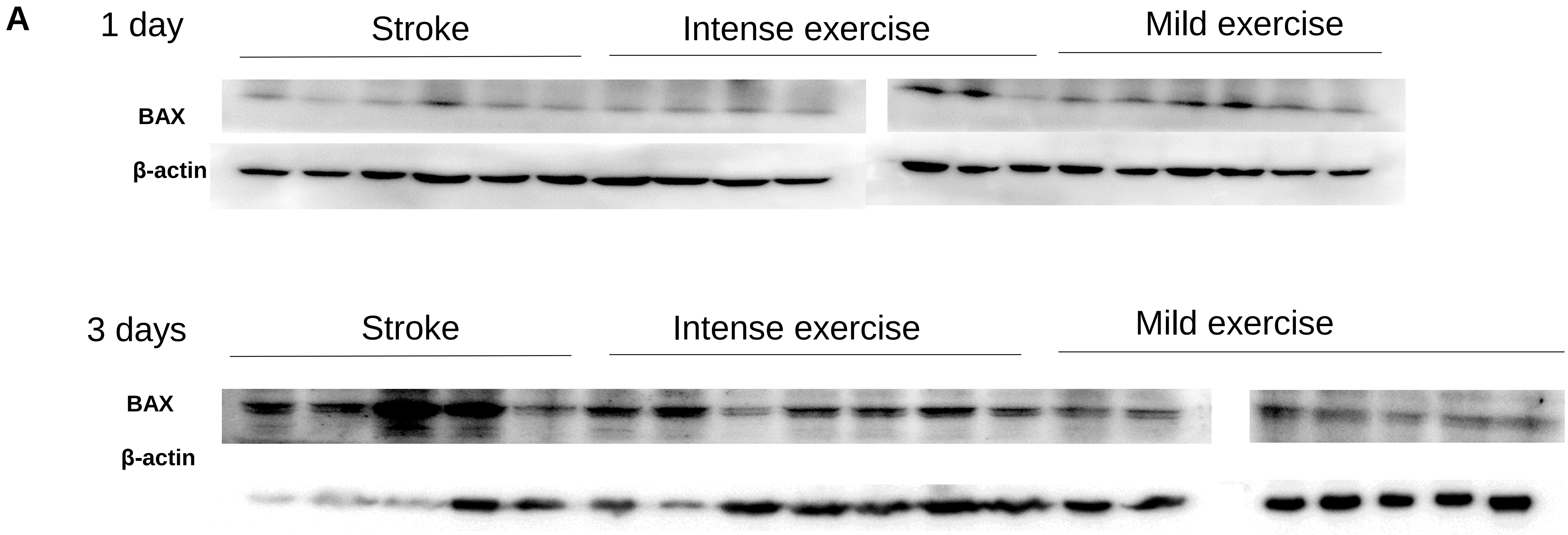
A
1 day
Stroke
Intense exercise
Mild exercise
BAX
β-actin
3 days
Stroke
Intense exercise
Mild exercise
BAX
β-actin

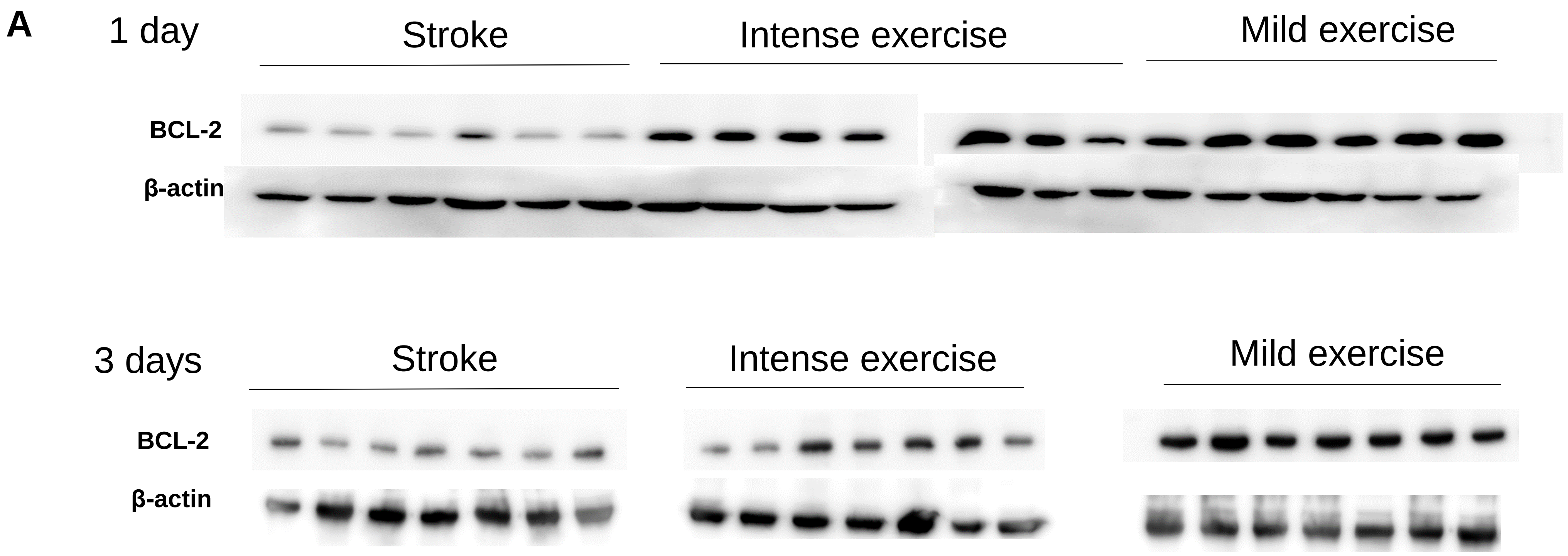
A
1 day
Stroke
Intense exercise
Mild exercise
BCL-2
β-actin
3 days
Stroke
Intense exercise
Mild exercise
BCL-2
β-actin

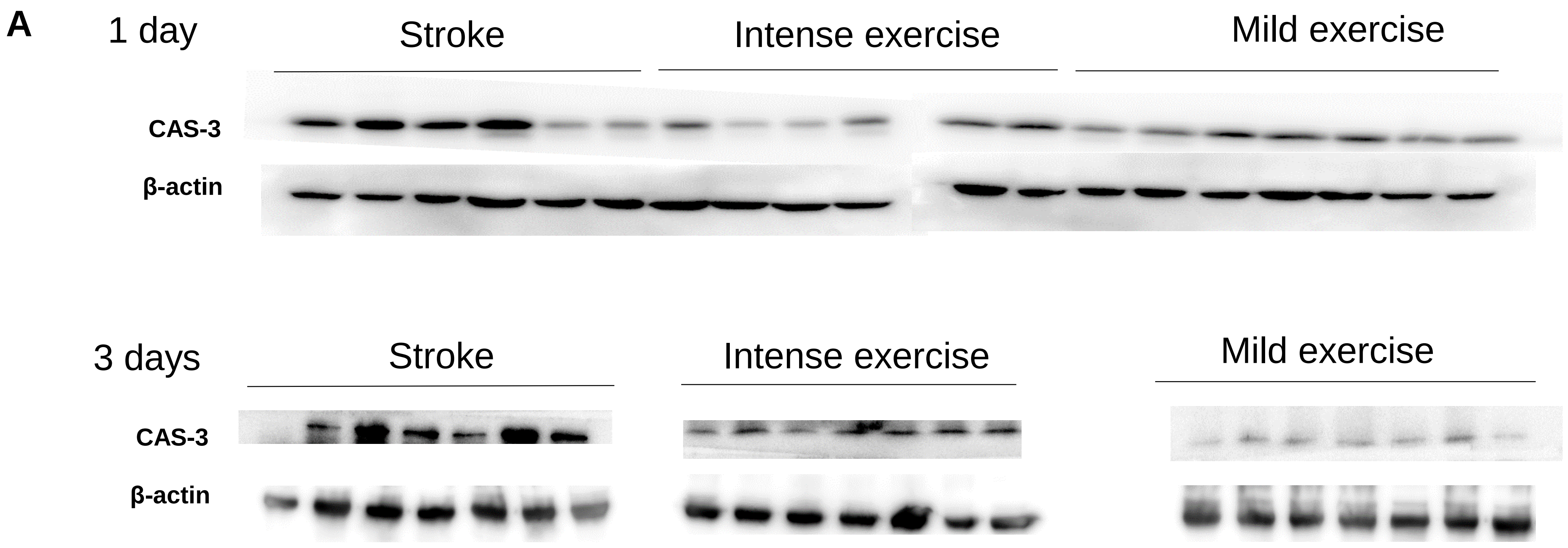
A
1 day
Stroke
Intense exercise
Mild exercise
CAS-3
β-actin
3 days
Stroke
Intense exercise
Mild exercise
CAS-3
β-actin

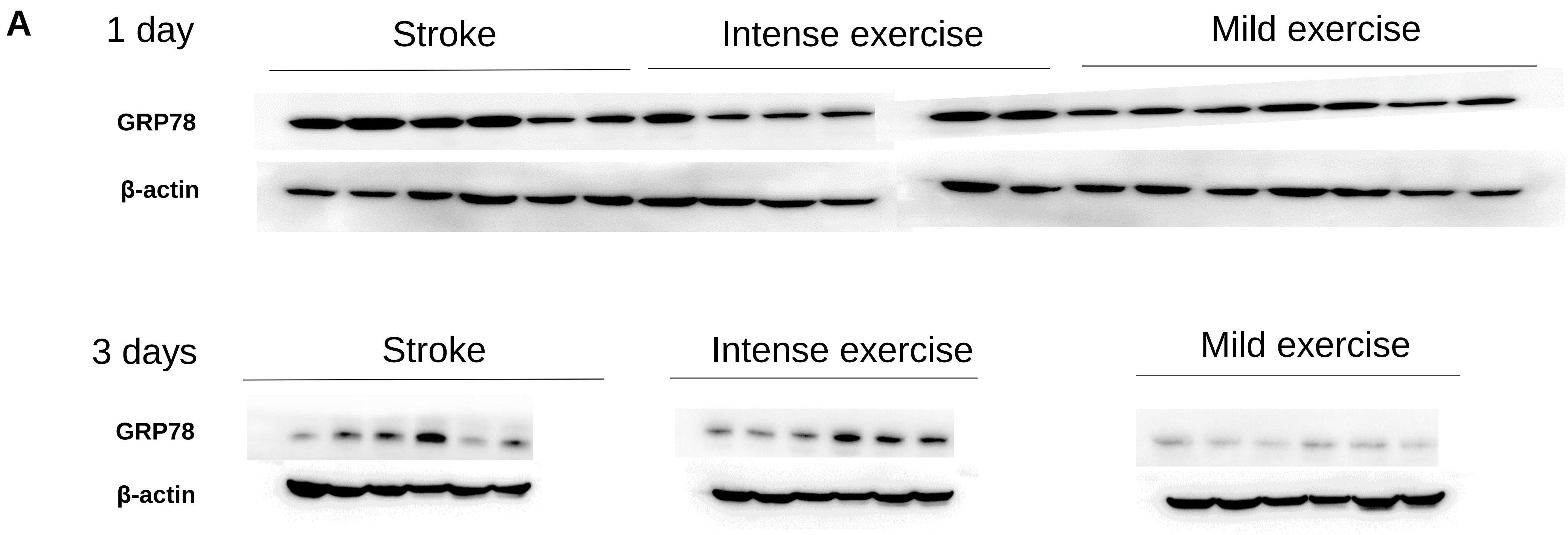
A
1 day
Stroke
Intense exercise
Mild exercise
GRP78
β-actin
3 days
Stroke
Intense exercise
Mild exercise
GRP78
β-actin

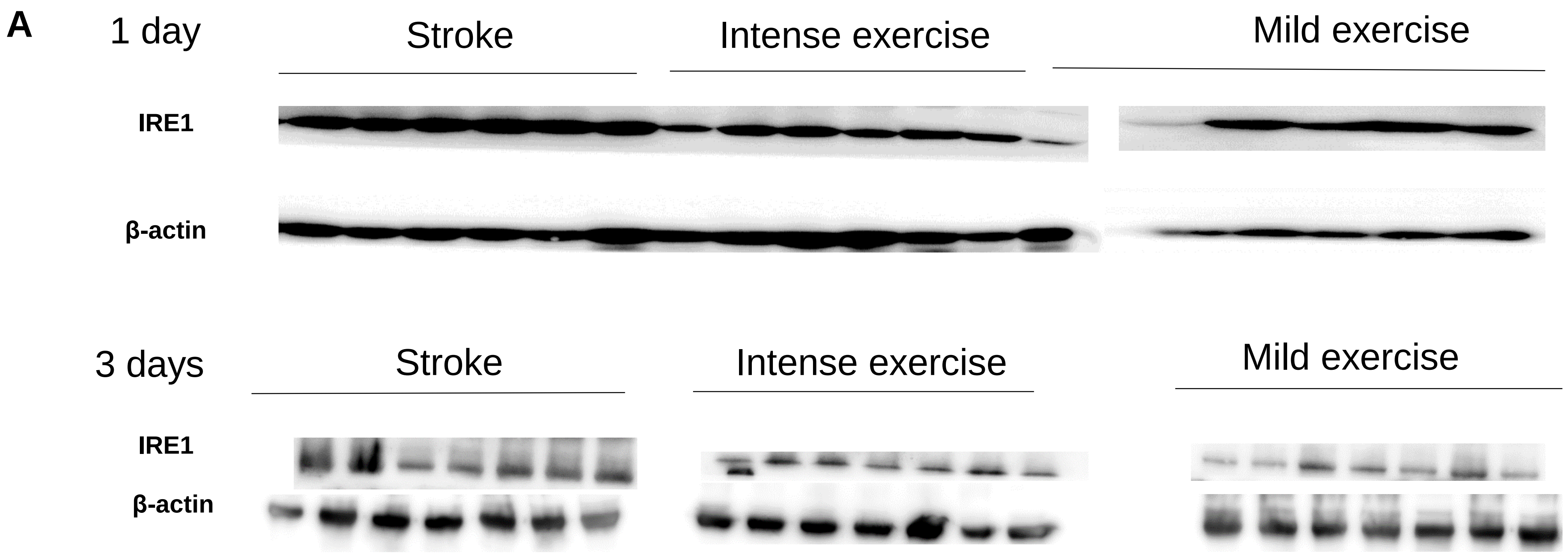
A
1 day
Stroke
Intense exercise
Mild exercise
IRE1
β-actin
3 days
Stroke
Intense exercise
Mild exercise
IRE1
β-actin

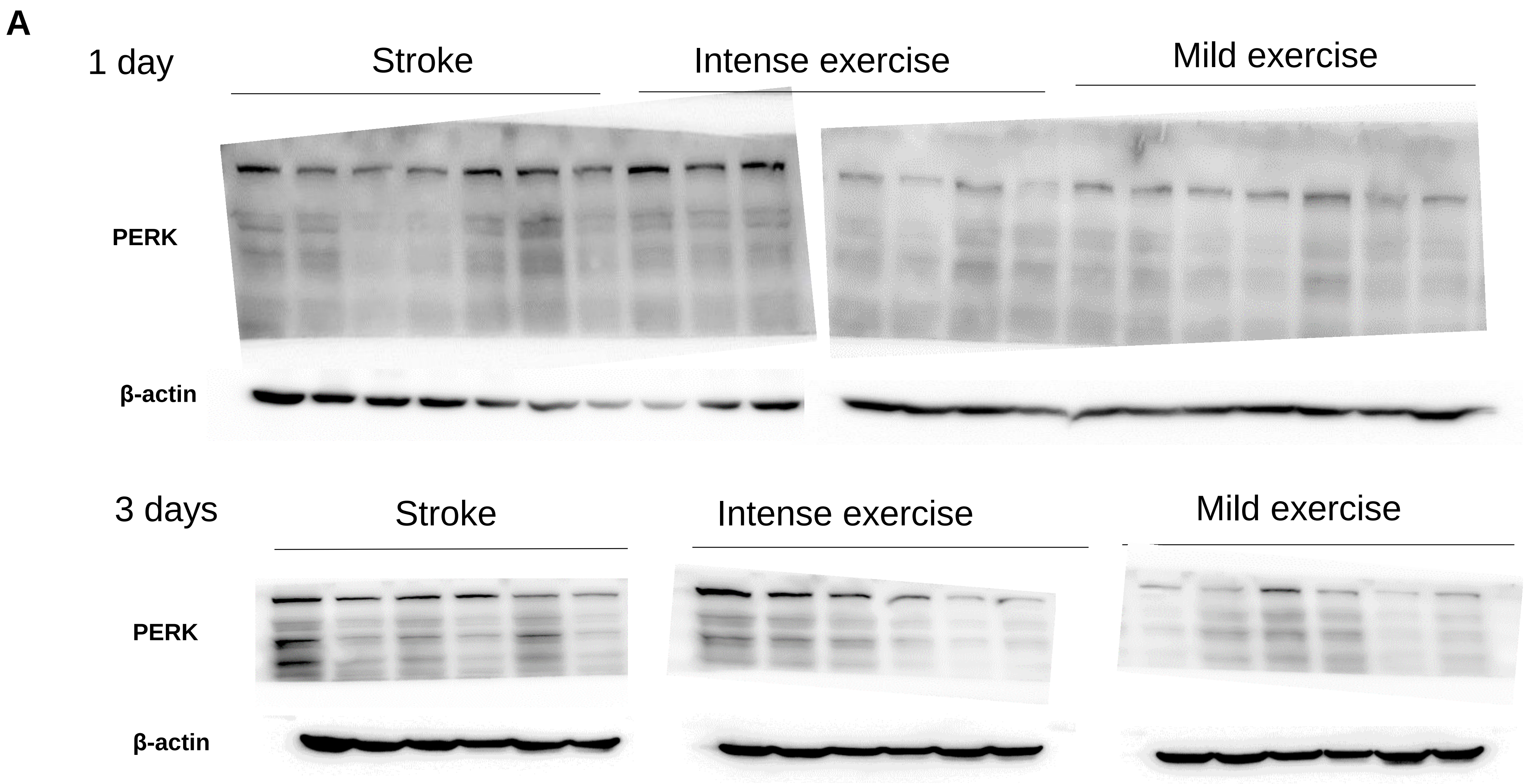
A
1 day
Stroke
Intense exercise
Mild exercise
PERK
β-actin
3 days
Stroke
Intense exercise
Mild exercise
PERK
β-actin

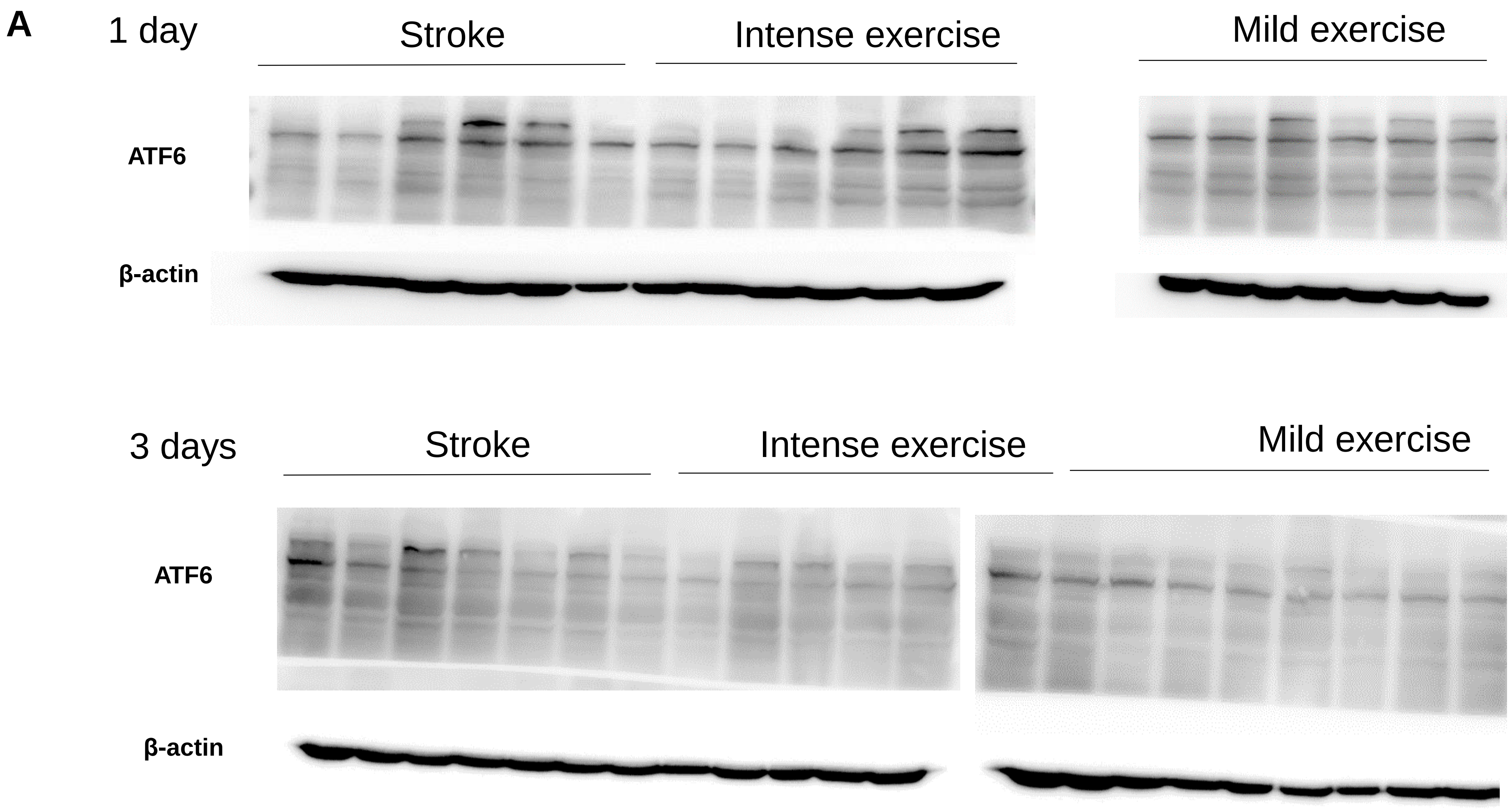
A
1 day
Stroke
Intense exercise
Mild exercise
ATF6
β-actin
3 days
Stroke
Intense exercise
Mild exercise
ATF6
β-actin

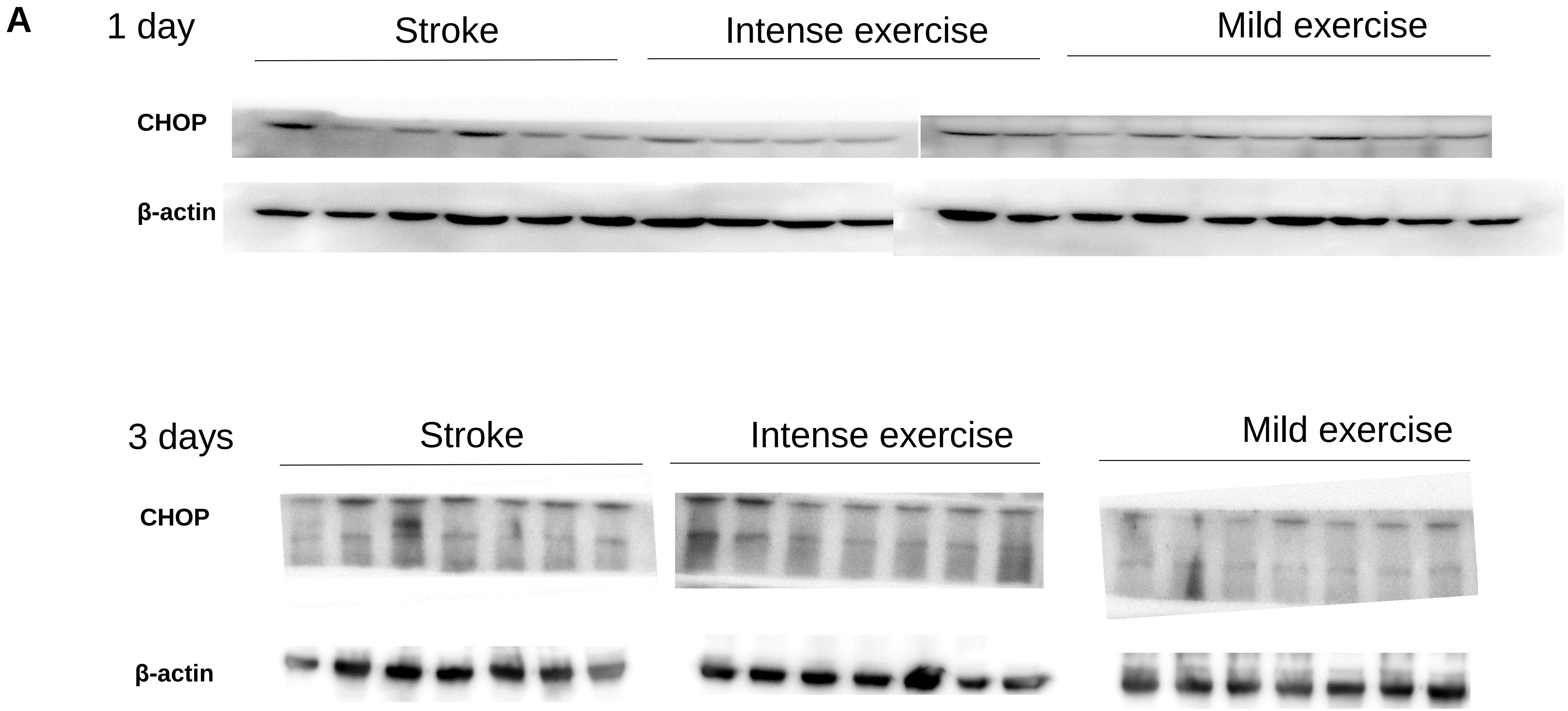
A
1 day
Stroke
Intense exercise
Mild exercise
CHOP
β-actin
3 days
Stroke
Intense exercise
Mild exercise
CHOP
β-actin

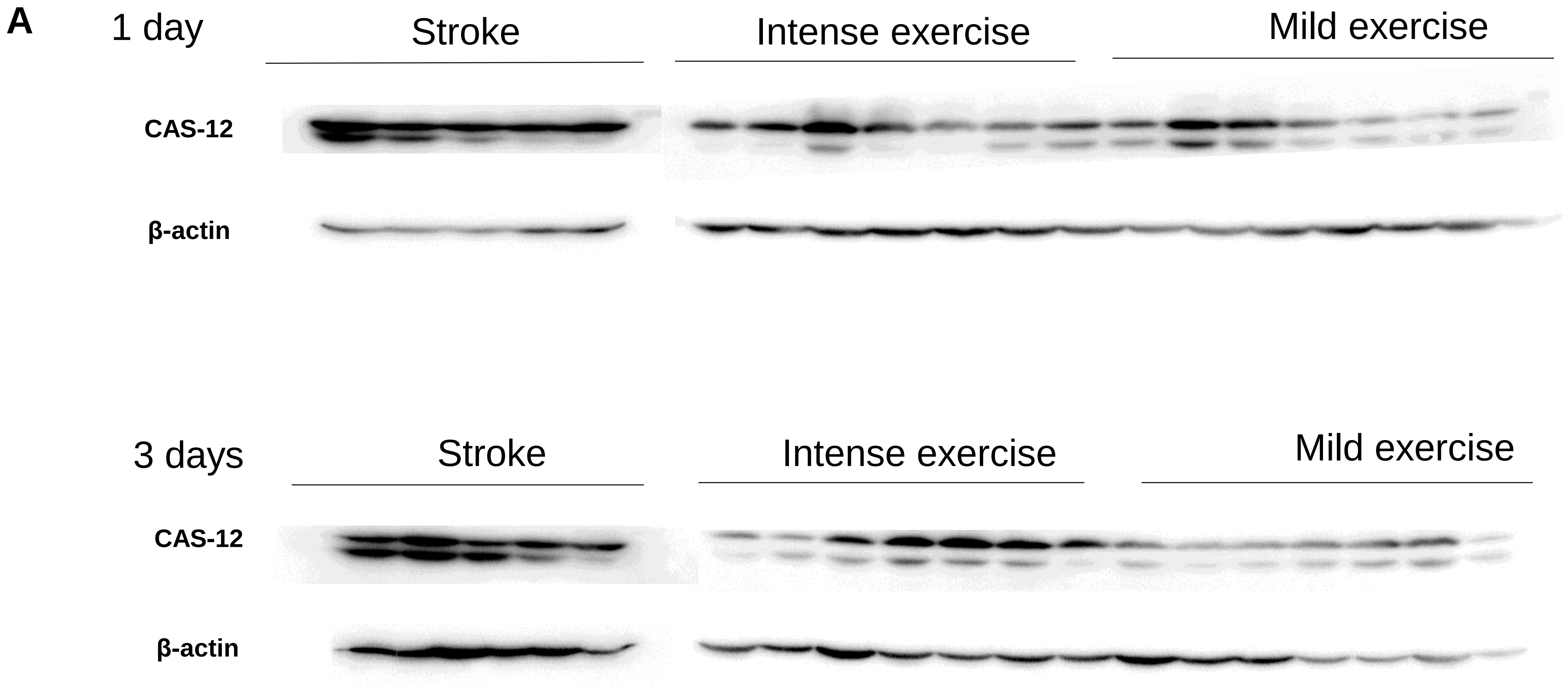
A
1 day
Stroke
Intense exercise
Mild exercise
CAS-12
β-actin
3 days
Stroke
Intense exercise
Mild exercise
CAS-12
β-actin

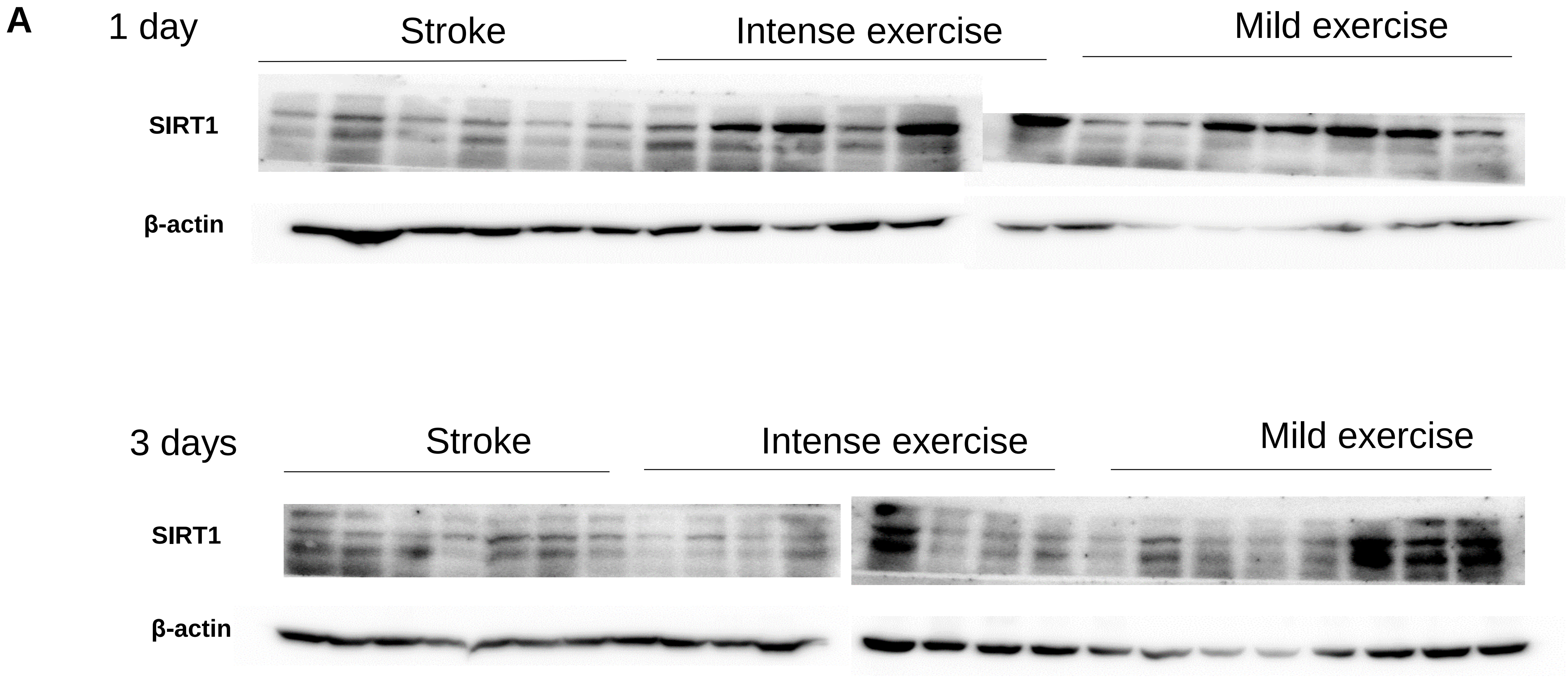
A
1 day
Stroke
Intense exercise
Mild exercise
SIRT1
β-actin
3 days
Stroke
Intense exercise
Mild exercise
SIRT1
β-actin
